# Effect of heating on disposal point of main edible oils available in Iran market

**DOI:** 10.1002/fsn3.3033

**Published:** 2022-08-29

**Authors:** Mehran Mohammadian Fazli, Hamid Zanganeh, Hassan Hassanzadazar

**Affiliations:** ^1^ Department of Environmental Health Engineering, School of Public Health Zanjan University of Medical Sciences Zanjan Iran; ^2^ Department of Food Safety and Hygiene, School of Public Health Zanjan University of Medical Sciences Zanjan Iran

**Keywords:** acid value, disposal point, edible oils, total polar compounds, vegetable oils

## Abstract

Deep frying is the most common method used for food preparation worldwide, which can lead to several chemical changes in used fat or oil in frying process including oxidation and polymerization. This study aimed to determine the effect of heating and different storage conditions on the disposal point of four types of the most common edible oils available in the Iranian market without and with the presence of food in terms of acid value (AV) and total polar compounds (TPC). AV and TPC of three types of marketed sunflower oil (especially for frying, cooking, solid vegetable) and ghee heated at 110, 150, 180, and 200°C with a portable oil meter DOM‐24 (ATAGO, Japan) were determined. They were measured without the presence of food at four different temperatures and four different times in five storage conditions and with the presence of frying food (chicken and potatoes) at 110°C for 20 min in three replicates. The results showed that the AV and TPC contents in the case of ghee were above national and international standards without the presence of food. Also, in the process of frying chicken and potato, the reusability of all the studied oils was not possible for the second time and reached the disposal point. The increase in the rate of AV and TPC of oils was: ghee (Kermanshahi oil) > liquid cooking oil > frying oil ≥ solid vegetable oil.

## Graphical abstract

TPC and AV exceeding rejection limit after one time repeated frying of food. The best desirable conditions for the storage of oils are storing in closed containers at 4C. Stability rate to food frying is: Ghee oil Cooking liquid oil Frying oil Solid vegetable oil.

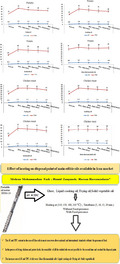

## 1 INTRODUCTION

Frequent use of edible oils for frying in food preparation and distribution centers, restaurants, confectioneries, and other food supply centers is a matter of concern for specialists in nutrition, food safety, and environmental health due to threat to consumer health. Therefore, inspection and monitoring of such centers using tools to identify harmful factors created during the food processing is on the agenda of health authorities of community. Unfortunately, some operators of restaurants, sandwich shops, and confectioneries threaten consumer health and safety due to improper use and reuse of heated edible oils (Farrokhzadeh et al., 2017; Hassanzadazar et al., 2018).

Early assessment of the heated oils' quality is an important step in implementing health and food safety programs (Song et al., 2017). Chemical analysis of heated oils in 35 countries in 2005–2006 showed that high density and the content of fatty acids especially trans isomers in oils cause adverse biological effects on humans (Ahmadi et al., 2018). The spontaneous oxidation and spoilage in these compounds takes place through a free radical chain mechanism that causes problems not only in the edible oils and food industry, but also in the consumers, which leads to adverse effects on the lungs, heart, liver, kidneys, eyes, blood, skin, muscles, and also accelerate the aging process (Neha et al., 2019). Carboxylic acids, aldehydes, straight chain alkanes, alcohols, alkadienes, and aromatic compounds are the main thermal degradation products of vegetable oils (Gomna et al., 2019).

Oil structure, heating time, temperature, and atmosphere type are the most important factors that have the greatest effects on the stability of edible oils during heating process (Gomna et al., 2019). Chemical reactions such as oxidation, hydrolysis, and polymerization of unsaturated fatty acids change the oil composition (Szabo et al., 2022). The content of polyunsaturated and saturated fatty acids of vegetable oils usually decreases and increases, respectively, during heating process (Szabo et al., 2022). The total polar compounds (TPC), such as decomposed products, nonhighly oxidized derivatives, polymeric and cyclic materials, and oil‐soluble compounds of fried foods, increase significantly during deep frying of edible oils (; Sanibal & Mancini Filho, 2004). According to the Iranian National Standard, the maximum content of TPC is 25% (Ghobadi et al., 2018).

During heating of edible oils an increase in the acid value (AV) occurs which is due to the hydrolysis of triacylglycerols in the oil. Since the rate of free fatty acids oxidation is higher than the oxidation of fatty acids involved in the triacylglycerols' structure, the lower content of free fatty acids leads to decrease in AV and higher oil stability. According to Codex Standard No. 210, before heating, the maximum AV of virgin and refined vegetable oils is 0.3% and 0.1%, respectively (Romero et al., 1998). Edible oils used for food frying or cooking goes to the disposal stage and become unusable when their TPC content and the hydrolysis of triacylglycerols (AV) reach over the maximum limits (Anonymous, 1999).

Changes that occur in used oils during cooking and deep frying can affect the nutritional quality of the food. The peroxide value, dimer and polymer contents, and free fatty acid increase in the frying oil (Sanibal & Mancini Filho, 2004). Several studies have been conducted in the field of increasing undesirable compounds in the oils used in cooking that mentioned endangering the health of the community. In a study by Jahed Khaniki et al. (2018), conducted in Tehran, Iran, on the oils consumed by fast food centers, 62.5% of the samples were unusable due to high peroxide content. In other studies, De Alzaa et al. (2018) showed that the production of polar compounds in the edible oils with the lower smoke point and oxidative stability was higher. The results of researches conducted by Ghobadi et al. (2018) and Ng et al. (2014) showed that the peroxide value of edible oils increases with increasing temperature and heating time.

There are several questions in the community with no enough scientific information about the usage frequency of an oil sample for food frying or cooking, storage of oil samples after heating, appropriate temperature for oil heating, storage condition of oil samples (storage in open door or close door containers or in environment temperature or refrigerators) under conventional household conditions. According to the literature review, there are no studies conducted to investigate the disposal point of all types of heated oils based on the AV and TPC in the presence or absence of food. Therefore, this study aimed to determine the effect of heating and different storage conditions on the disposal point of four types of the most common edible oils available in the Iranian market without and with the presence of food in terms of AV and TPC.

## 2 MATERIALS AND METHODS

### 2.1 Materials

Digital plate heater (Pars Azma, Iran) was used to heat the oils. A frying oil monitor DOM‐24 (ATAGO, Japan) was used to measure AV and TPC in oil samples. The samples of three types of marketed sunflower oil (especially for frying, cooking, and semihydrogenated) and ghee were provided in Zanjan market, Iran.

### 2.2 Calibration of devices

Before preparing the samples and starting the tests, all used devices were calibrated to make the measurements with high accuracy and precision. Frying oil monitor DOM‐24 (ATAGO, Japan) has been prepared and approved by the Health and Medical Education Ministry of Iran. This device has been adapted to Iran National Standard No. 4152 to determine the disposal point of edible oils used in food preparation and distribution centers (ISIRI, 1999).

### 2.3 Preparation of samples

Three types of the most common sunflower edible oils (frying, cooking, and semisolid) were obtained from food supply market at the same time in the same stores and with the same expiry date and one type of ghee was obtained from traditional dairy retailers. At first, three batches of each edible oil containing 1 L oil were prepared for measurement in triplicate. Then, AV and TPC of 1 L (each batch) of all provided samples were measured. After that, each 1 L container was divided into four equal containers. The prepared oils were heated and tested at four different temperatures (110, 150, 180, and 200°C) and four times (5, 10, 15, and 20 min) with three replicates. All measurements were performed on the same temperature, but four different times. After heating, oil samples were stored in four specific conditions (container number 1: at room temperature in an open door container for 6 h; container number 2: at room temperature in a closed door container for 6 h; container number 3: kept in the refrigerator in an open door container for 6 h; container number 4: in the refrigerator in a closed door container for 6 h) and the desired parameters were measured and recorded again after 6 h. The AV and TPC were also measured in all four types of oil samples used for frying of two foods (chicken meat and potato) at a temperature of 110°C and heating time of 20 min in all the above conditions. The oil temperature drops as soon as it comes in contact with the food which returns quickly to initial temperature, therefore, the measurement of AV and TPC will be taken when the oil temperature reaches again to 110°C. The time measurement started when food was put in the oil ignoring the temperature drop. The disposal point of oil samples was when the AV and TPC reach to ≥1% and ≥25%, respectively.

### 2.4 Statistical analysis

The average and standard deviation of the obtained AV and TPC percentage and graphs were calculated and drawn using Excell software. All measurements were reported in one and two decimal places for means and standard deviations, respectively. Statistically significant difference between the samples in different conditions of heating and storage was determined by SPSS software version 26 (St. Louis, IL, USA) using general linear model (univariate analysis of variance) test. All tests were performed in triplicate, in other word; all measurements were conducted on the three provided batches of each edible oil.

## 3 RESULTS AND DISCUSSION

**FIGURE 1:**
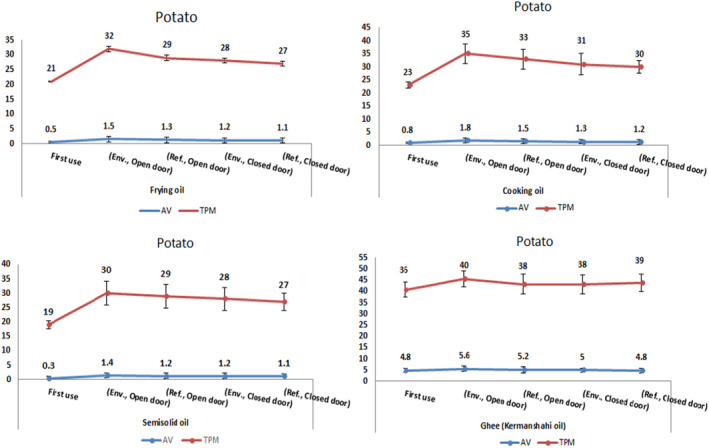
AV and TPC changes in heated oils containing potato at 110°C for 20 min in five conditions. Abbreviations: AV, acid value; Env., environment; Ref., refrigerator; TPC, total polar compounds

**FIGURE 2:**
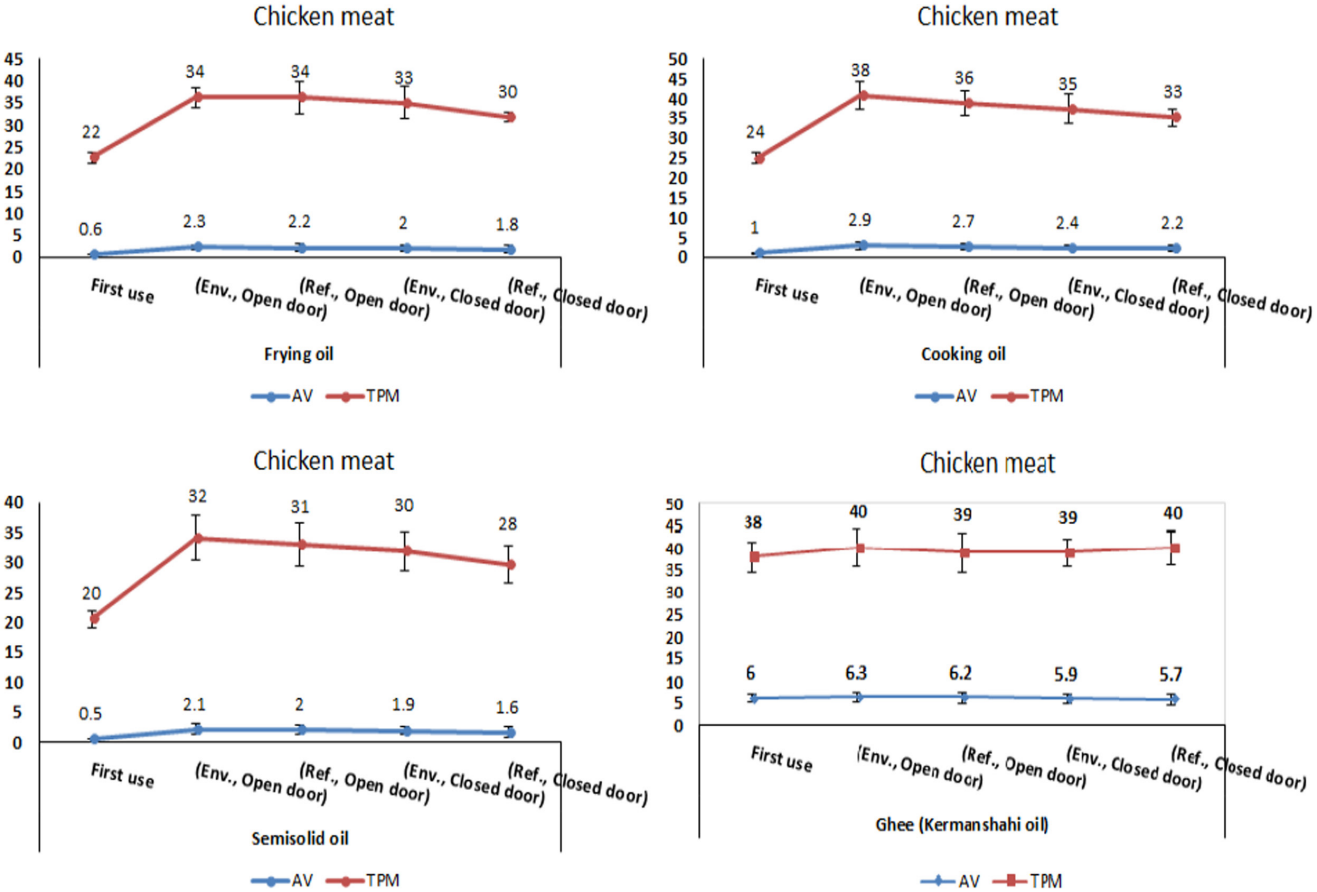
AV and TPC changes in heated oils containing chicken meat at 110°C for 20 min in five conditions. Abbreviations: AV, acid value; Env., environment; Ref., refrigerator; TPC, total polar compounds

Evaluation of AV and TPC of desired oils under considered conditions including oil type, heating temperature, and their storage condition revealed that heating of oils without the presence of food in all conditions leads to ascending manner of AV and TPC averages but in a healthy limit (*p* > .05) except the ghee samples with significant difference between mean values of fresh unheated and heated samples (*p* < .05) (Tables 1, 2, 3). Frying the food samples (potato and chicken meat) in the selected oils at 110°C for 20 min in designated conditions showed that all tested oils reached to disposal point in the presence of food except for the first use of three oils (Figures 1, 2). The ghee (once oil reached the given temperature) had reached to the disposal point at the beginning of the frying process based on AC and TPC measurements (Tables 1, 2, 3). The oils used for frying the chicken meat always showed a higher amount of AV and TPC than potatoes (Figures 1, 2). Chicken released fat into the frying medium, but potato absorb oil during frying; that can be a main reason to reach the oils used during the frying of chicken to disposal point more rapid than the oils used for frying potato (Goburdhun et al., 2008).

**TABLE 1:** Initial acid value (AV) and total polar compounds (TPC) (%) contents of fresh provided samples of edible oils at room temperature

Edible oil type	Frying oil	Cooking oil	Semisolid oil	Ghee
AV	0.1	0.1	0.1	0.5
TPC	1.1	1	0.9	15

**TABLE 2:** Mean ± standard deviation of measured acid value of heated oils without food samples

Measurement condition	Edible oil type	5 min				10 min				15 min				20 min			
		110°C	150°C	180°C	200°C	110°C	150°C	180°C	200°C	110°C	150°C	180°C	200°C	110°C	150°C	180°C	200°C
First use	Frying oil	0.1 ± 0.01	0.1 ± 0.02	0.1 ± 0.06	0.2 ± 0.06	0.1 ± 0.01	0.1 ± 0.03	0.1 ± 0.05	0.2 ± 0.06	0.3 ± 0.06	0.1 ± 0.04	0.1 ± 0.05	0.3 ± 0.06	0.3 ± 0.06	0.2 ± 0.06	0.4 ± 0.06	0.4 ± 0.04
	Cooking oil	0.1 ± 0.01	0.2 ± 0.06	0.2 ± 0.06	0.3 ± 0.06	0.1 ± 0.01	0.2 ± 0.02	0.3 ± 0.0.9	0.4 ± 0.04	0.2 ± 0.04	0.2 ± 0.05	0.4 ± 0.06	0.4 ± 0.06	0.2 ± 0.06	0.3 ± 0.05	0.5 ± 0.06	0.5 ± 0.06
	Semisolid oil	0.1 ± 0.01	0.1 ± 0.01	0.1 ± 0.06	0.2 ± 0.04	0.1 ± 0.01	0.1 ± 0.03	0.2 ± 0.05	0.2 ± 0.06	0.1 ± 0.01	0.2 ± 0.04	0.2 ± 0.06	0.2 ± 0.06	0.1 ± 0.01	0.2 ± 0.06	0.3 ± 0.06	0.3 ± 0.04
	Ghee	1.2 ± 0.31	2.1 ± 0.11	2.2 ± 0.31	2.3 ± 0.31	2.2 ± 0.52	2.2 ± 0.31	2.3 ± 0.31	2.8 ± 0.33	2.1 ± 0.31	2.7 ± 0.32	2.8 ± 0.32	3.1 ± 0.34	2.7 ± 0.32	3.3 ± 0.22	3.3 ± 0.33	3.7 ± 0.34
Environment (open door)	Frying oil	0.1 ± 0.01	0.1 ± 0.06	0.2 ± 0.06	0.3 ± 0.06	0.2 ± 0.06	0.2 ± 0.06	0.3 ± 0.06	0.4 ± 0.04	0.2 ± 0.04	0.3 ± 0.05	0.3 ± 0.06	0.4 ± 0.04	0.3 ± 0.06	0.3 ± 0.06	0.4 ± 0.06	0.5 ± 0.06
	Cooking oil	0.1 ± 0.06	0.2 ± 0.06	0.3 ± 0.04	0.4 ± 0.06	0.2 ± 0.06	0.3 ± 0.04	0.3 ± 0.06	0.5 ± 0.06	0.3 ± 0.06	0.3 ± 0.04	0.4 ± 0.06	0.5 ± 0.06	0.3 ± 0.06	0.4 ± 0.06	0.6 ± 0.06	0.6 ± 0.06
	Semisolid oil	0.1 ± 0.06	0.2 ± 0.06	0.2 ± 0.06	0.3 ± 0.04	0.1 ± 0.06	0.2 ± 0.10	0.3 ± 0.06	0.4 ± 0.06	0.1 ± 0.01	0.2 ± 0.09	0.3 ± 0.08	0.4 ± 0.04	0.2 ± 0.01	0.3 ± 0.06	0.4 ± 0.10	0.5 ± 0.10
	Ghee	2.2 ± 0.22	2.3 ± 0.31	2.7 ± 0.31	3 ± 0.41	2.7 ± 0.31	2.8 ± 0.31	3.2 ± 0.33	3.3 ± 0.33	3 ± 0.11	3.5 ± 0.51	3.7 ± 0.33	3.8 ± 0.34	3.3 ± 0.31	3.7 ± 0.32	3.8 ± 0.33	4.3 ± 0.34
Environment (close door)	Frying oil	0.1 ± 0.01	0.1 ± 0.06	0.3 ± 0.06	0.3 ± 0.06	0.2 ± 0.06	0.2 ± 0.03	0.3 ± 0.06	0.3 ± 0.06	0.2 ± 0.01	0.3 ± 0.06	0.3 ± 0.06	0.4 ± 0.06	0.3 ± 0.06	0.3 ± 0.06	0.4 ± 0.10	0.4 ± 0.06
	Cooking oil	0.2 ± 0.01	0.3 ± 0.06	0.4 ± 0.06	0.4 ± 0.04	0.2 ± 0.06	0.3 ± 0.06	0.4 ± 0.05	0.5 ± 0.06	0.3 ± 0.01	0.3 ± 0.04	0.5 ± 0.06	0.5 ± 0.06	0.3 ± 0.04	0.4 ± 0.06	0.5 ± 0.06	0.6 ± 0.06
	Semisolid oil	0.1 ± 0.01	0.1 ± 0.06	0.1 ± 0.06	0.2 ± 0.06	0.1 ± 0.01	0.1 ± 0.03	0.2 ± 0.06	0.2 ± 0.06	0.1 ± 0.06	0.2 ± 0.04	0.2 ± 0.05	0.3 ± 0.10	0.1 ± 0.08	0.2 ± 0.08	0.3 ± 0.08	0.3 ± 0.10
	Ghee	1.7 ±0.31	1.8 ±0.31	2.2 ± 0.33	2.5 ± 0.31	2.2 ± 0.31	2.5 ± 0.51	3.3 ± 0.31	3.0 ± 0.39	2.7 ± 0.41	3.0 ± 0.31	3.2 ± 0.32	3.3 ± 0.32	3.0 ± 0.34	3.2 ± 0.33	3.5 ± 0.34	3.7 ± 0.34
Refrigerator (open door)	Frying oil	0.1 ± 0.01	0.1 ± 0.06	0.2 ± 0.06	0.2 ± 0.06	0.1 ± 0.01	0.1 ± 0.06	0.3 ± 0.06	0.3 ± 0.06	0.1 ± 0.06	0.2 ± 0.04	0.3 ± 0.06	0.3 ± 0.06	0.2 ± 0.04	0.2 ± 0.05	0.3 ± 0.10	0.4 ± 0.10
	Cooking oil	0.1 ± 0.06	0.3 ± 0.04	0.3 ± 0.06	0.4 ± 0.10	0.2 ± 0.06	0.3 ± 0.06	0.4 ± 0.10	0.4 ± 0.06	0.2 ± 0.06	0.3 ± 0.06	0.3 ± 0.06	0.5 ± 0.06	0.3 ± 0.06	0.4 ± 0.10	0.5 ± 0.10	0.5 ± 0.06
	Semisolid oil	0.1 ± 0.01	0.1 ± 0.06	0.2 ± 0.04	0.3 ± 0.06	0.1 ± 0.06	0.1 ± 0.04	0.2 ± 0.06	0.3 ± 0.06	0.1 ± 0.01	0.1 ± 0.04	0.2 ± 0.06	0.3 ± 0.06	0.1 ± 0.01	0.2 ± 0.06	0.3 ± 0.06	0.3 ± 0.06
	Ghee	2.0 ± 0.31	1.8 ±0.31	2.3 ±0.31	2.5 ± 0.06	2.2 ±0.31	2.5 ± 0.51	2.8 ± 0.31	3.0 ± 0.44	2.3 ± 0.51	3.2 ± 0.31	3.3 ± 0.32	3.3 ± 0.31	3.0 ± 0.33	3.3 ± 0.31	3.5 ± 0.51	4.0 ± 0.31
Refrigerator (close door)	Frying oil	0.1 ± 0.05	0.1 ± 0.06	0.2 ± 0.06	0.2 ± 0.06	0.1 ± 0.01	0.2 ± 0.06	0.2 ± 0.06	0.3 ± 0.06	0.1 ± 0.01	0.1 ± 0.06	0.2 ± 0.06	0.3 ± 0.10	0.2 ± 0.06	0.2 ± 0.06	0.3 ± 0.10	0.3 ± 0.10
	Cooking oil	0.1 ± 0.01	0.2 ± 0.06	0.3 ± 0.06	0.3 ± 0.06	0.2 ± 0.06	0.3 ± 0.06	0.3 ± 0.06	0.4 ± 0.10	0.2 ± 0.04	0.3 ± 0.06	0.4 ± 0.06	0.5 ± 0.10	0.3 ± 0.06	0.4 ± 0.06	0.5 ± 0.06	0.5 ± 0.10
	Semisolid oil	0.1 ± 0.01	0.1 ± 0.02	0.1 ± 0.06	0.2 ± 0.06	0.1 ± 0.06	0.2 ± 0.02	0.2 ± 0.09	0.3 ± 0.10	0.1 ± 0.06	0.1 ± 0.06	0.2 ± 0.06	0.3 ± 0.10	0.1 ± 0.06	0.2 ± 0.06	0.2 ± 0.06	0.3 ± 0.04
	Ghee	1.3 ± 0.61	2.0 ± 0.11	2.3 ± 0.31	2.5 ± 0.51	2.0 ± 0.10	2.2 ± 0.31	2.5 ± 0.51	2.7 ± 0.31	2.7 ± 0.31	2.8 ± 0.31	3.0 ± 0.10	3.3 ± 0.31	2.8 ± 0.31	3.2 ± 0.31	3.3 ± 0.10	3.7 ± 0.31

**TABLE 3:** Mean ± standard deviation of measured total polar compounds of heated oils without food samples

Measurement condition	Edible oil type	5 min				10 min				15 min				20 min			
		110°C	150°C	180°C	200°C	110°C	150°C	180°C	200°C	110°C	150°C	180°C	200°C	110°C	150°C	180°C	200°C
First use	Frying oil	2.3 ± 0.60	2.7 ± 0.60	4 ± 1.00	4.3 ± 0.60	2.3 ± 0.60	3.3 ± 0.60	4 ± 1.00	5.0 ± 1.00	3.3 ± 0.60	4.3 ± 0.60	4.7 ± 0.60	6.0 ± 0.01	4.0 ± 0.01	4.7 ± 0.60	5.7 ± 0.60	7.0 ± 1.00
	Cooking oil	3.0 ± 0.01	3.3 ± 0.60	4.7 ± 0.60	5.3 ± 0.60	4.3 ± 0.60	5.7 ± 0.60	6.3 ± 0.60	7.3 ± 0.60	4.7 ± 0.60	6.0 ± 0.01	6.7 ± 0.60	7.3 ± 0.60	5.7 ± 0.60	7.0 ± 1.00	7.7 ± 0.60	9.0 ± 1.00
	Semisolid oil	1.7 ± 0.60	2.3 ± 0.61	3.0 ± 0.01	3.0 ± 0.01	2.0 ± 0.01	2.7 ± 0.60	3.3 ± 0.60	4.0 ± 0.01	2.0 ± 0.01	3.3 ± 0.60	3.7 ± 0.61	5.0 ± 0.01	3.0 ± 0.01	4.3 ± 0.60	5.0 ± 0.01	5.7 ± 0.60
	Ghee	21.2 ± 1.00	23.3 ± 2.00	25.3 ± 1.00	26.5 ± 1.00	23.1 ± 2.00	24.2 ± 1.00	25.3 ± 1.00	27.5 ± 1.00	24.2 ± 2.00	25.2 ± 1.00	26.4 ± 1.00	28.4 ± 1.00	25.5 ± 2.00	27.5 ± 1.00	28.2 ± 1.00	30.1 ± 2.00
Environment (open door)	Frying oil	3.3 ± 0.60	4.2 ± 0.01	5.3 ± 0.60	6.3 ± 0.61	3.3 ± 0.60	4.7 ± 0.60	6 ± 0.01	7.0 ± 0.01	4.7 ± 0.60	5.3 ± 0.60	6.0 ± 0.01	8.0 ± 0.01	4.7 ± 0.60	6.0 ± 0.01	8.0 ± 1.00	10.0 ± 1.00
	Cooking oil	5.3 ± 0.60	6.7 ± 0.60	7.3 ± 0.60	8.7 ± 0.60	8.3 ± 0.60	9.3 ± 1.5	11.0 ± 1.00	13.0 ± 1.00	10.0 ± 1.00	11.7 ± 2.3	13.0 ± 1.00	14.0 ± 0.01	10.3 ± 0.60	11.0 ± 1.00	5.3 ± 0.60	15.3 ± 0.60
	Semisolid oil	3.7 ± 1.20	4.0 ± 1.00	4.3 ± 0.60	6.0 ± 1.00	4.0 ± 1.00	4.7 ± 0.60	5.7 ± 0.60	6.7 ± 0.60	5.0 ± 1.00	5.7 ± 1.20	7.0 ± 1.00	7.3 ± 0.60	5.0 ± 0.01	6.3 ± 0.60	8.0 ± 1.00	8.7 ± 1.20
	Ghee	25.0 ± 2.00	27.0 ± 1.00	28.0 ± 1.00	30.0 ± 2.00	26.0 ± 1.00	28.0 ± 1.00	29.0 ± 1.00	31.0 ± 2.00	27.0 ± 1.00	28.0 ± 3.00	30.0 ± 3.00	33.0 ± 2.00	29.0 ± 1.00	31.0 ± 2.00	33.0 ± 3.00	36.0 ± 2.00
Environment (close door)	Frying oil	3.3 ± 0.60	3.7 ± 0.60	4.3 ± 0.60	4.7 ± 0.60	3.3 ± 0.60	4.3 ± 0.60	5.0 ± 0.01	5.3 ± 0.60	4.3 ± 0.60	5.0 ± 1.00	6.0 ± 0.01	6.3 ± 0.60	5.0 ± 1.00	6.0 ± 0.01	7.7 ± 0.60	8.3 ± 0.60
	Cooking oil	4.3 ± 0.60	5.7 ± 0.60	5.7 ± 0.60	7.0 ± 1.00	6.0 ± 1.00	8.0 ± 1.00	9.3 ± 1.50	11.0 ± 1.00	5.0 ± 1.00	8.7 ± 2.10	10.0 ± 1.70	12.3 ± 0.60	9.0 ± 1.00	10.3 ± 1.50	12.0 ± 1.00	1.4 ± 1.00
	Semisolid oil	3.3 ± 0.60	3.7 ± 0.60	4.7 ± 0.60	5.3 ± 0.60	3.7 ± 0.60	4.3 ± 0.60	5.3 ± 0.60	5.3 ± 0.60	4.3 ± 0.60	5.3 ± 0.60	6.0 ± 1.00	6.7 ± 0.60	5.0 ± 0.01	6.0 ± 1.00	7.3 ± 1.20	8.0 ± 1.00
	Ghee	24.0 ± 2.00	26.0 ± 1.00	27.0 ± 1.00	28.0 ± 2.00	25.0 ± 1.00	26.0 ± 1.00	28.0 ± 1.00	29.0 ± 2.00	25.0 ± 1.00	27.0 ± 1.00	28.0 ± 1.00	31.0 ± 2.00	27.0 ± 1.00	29.0 ± 1.00	31.0 ± 2.00	33.0 ± 2.00
Refrigerator (open door)	Frying oil	3.0 ± 0.01	3.7 ± 0.60	4.3 ± 0.60	4.7 ± 0.60	3.3 ± 0.60	3.7 ± 0.60	4.3 ± 0.60	5.0 ± 0.01	3.3 ± 0.60	4.7 ± 0.60	5.0 ± 0.01	6.3 ± 0.60	4.3 ± 0.60	4.7 ± 0.60	6.0 ± 0.60	8.0 ± 1.00
	Cooking oil	3.3 ± 0.60	4.7 ± 0.60	5.3 ± 0.60	5.7 ± 0.60	5.3 ± 1.50	6.7 ± 1.20	8.0 ± 1.00	9.0 ± 2.00	8.0 ± 2.00	9.7 ± 1.50	11.7 ± 1.50	12.3 ± 1.20	7.0 ± 0.01	7.7 ± 0.60	11.0 ± 1.00	12.3 ± 0.60
	Semisolid oil	3.3 ± 0.60	3.7 ± 0.60	4.3 ± 0.60	5.0 ± 0.01	3.7 ± 0.60	5.0 ± 0.01	5.0 ± 0.01	5.3 ± 0.60	3.3 ± 0.60	5.0 ± 0.01	5.7 ± 0.60	6.0 ± 0.01	4.0 ± 1.00	4.7 ± 0.60	5.3 ± 0.60	6.3 ± 0.60
	Ghee	23.0 ± 2.00	25.0 ± 1.00	26.0 ± 1.00	27.0 ± 1.00	24.0 ± 1.00	25.0 ± 1.00	27.0 ± 2.00	28.0 ± 2.00	24.0 ± 2.00	26.0 ± 1.00	27.0 ± 1.00	29.0 ± 2.00	26.0 ± 1.00	28.0 ± 1.00	30.0 ± 2.00	32.0 ± 2.00
Refrigerator (close door)	Frying oil	2.7 ± 0.60	3.0 ± 0.01	4.0 ± 1.00	5.0 ± 0.01	3.0 ± 0.01	3.7 ± 0.60	4.3 ± 0.60	5.0 ± 0.01	3.0 ± 0.01	4.7 ± 0.60	5.3 ± 0.60	5.3 ± 0.60	4.7 ± 0.60	5.3 ± 0.60	6.3 ± 1.00	8.0 ± 1.00
	Cooking oil	3.3 ± 0.60	4.0 ± 0.01	5.0 ± 0.60	5.7 ± 0.60	4.3 ± 0.60	6.0 ± 1.00	7.3 ± 1.20	9.0 ± 1.00	7.0 ± 1.00	8.7 ± 0.60	9.3 ± 0.60	11.0 ± 1.00	6.7 ± 0.60	8.0 ± 1.00	9.0 ± 0.00	11.0 ± 1.00
	Semisolid oil	2.0 ± 0.01	2.7 ± 0.60	4.0 ± 1.00	4.3 ± 0.60	2.3 ± 0.60	3.7 ± 0.60	4.3 ± 0.60	5.0 ± 1.00	3.3 ± 0.60	4.0 ± 0.01	5.3 ± 0.60	6.3 ± 0.60	3.3 ± 0.60	4.7 ± 0.60	6.0 ± 1.00	7.3 ± 0.60
	Ghee	22.0 ± 1.00	23.0 ± 2.00	25.0 ± 1.00	28.0 ± 1.00	24.0 ± 1.00	26.0 ± 1.00	27.0 ± 1.00	28.0 ± 2.00	25.0 ± 1.00	26.0 ± 1.00	28.0 ± 1.00	30.0 ± 1.00	27.0 ± 1.00	28.0 ± 1.00	30.0 ± 1.00	32.0 ± 2.00

There are several methods to prepare foods in home, food preparation centers, and food industries including frying and cooking, which lead to desirable color, flavor, viscosity, and texture. Deep frying is the most common method worldwide that can undergo chemical changes (oxidation, hydrolysis, and polymerization of unsaturated fatty acids) during heating in the presence of air and moisture (from food) (Szabo et al., 2022; Jurid et al., 2020). These chemical changes enhance with heating time and temperature and are accelerated by the presence of food. Heating of oils at high temperatures in the presence of oxygen can have detrimental oxidative effects. The oxygen in the atmosphere and the moisture in the frying food are mixed together and intensify the oxidation of the oil (Wang et al., 2021).

In this study, the effect of heating on the disposal point of three types of the most common sunflower oils available in the Iranian market and ghee (obtained from cow butter) was investigated by the determination of AV and TPC using the DOM‐24 oil monitor (ATAGO, Japan). Firestone (2007) showed that the determination of TPC as a qualitative indicator for edible oils, especially frying oils, has been accepted worldwide and in many developed countries the maximum accepted limit is 24%–27%. According to the National Standard of Iran, the maximum TPC and AV of oils are 25% and 1%, respectively (ISIRI, 1999).

The trend of changes measured in this study in independent variables without the presence of food (mean ± standard deviation of AV and TPC percentage) is demonstrated in Tables 1 and 2, as well as with the presence of food is shown in Figures 1 and 2. The results of Tables 1 and 2 showed that the increase in AV and percentage of TPC is directly related to the increase in heating time and temperature until reaching to the disposal point. Excessive heating in the presence of air causes oxidative changes in the unsaturated acyl groups in glycerides and other unsaturated components in oils and fats. These changes alter the nutritional properties of fats and lead to the formation of oxidized and polymerized compounds (Wang et al., 2021).

In general, many factors are effective in degrading and reaching the disposal point of the oil including temperature elevation, time and frequency of use, and maintaining condition that affect the AV and the percentage of TPC (Asokapandian et al., 2019; Song et al., 2017; Navab Daneshmand & Ghavami, 2012). Variance analysis of the results in this study showed significant differences of the changes of these variables. Oil types, time and temperature variation, open and close storage, and addition of food for frying to used oils are the major factors that can affect the AV and TPC contents (*p* < 0.05) (Tables 4, 5). As indicated in Tables 3 and 4, according to the obtained results, addition of food to oils for frying and oil types are the main factors that cause increase of AV and TPC contents. These findings were consistent with the research of Yu et al. (2018) and Song et al. (2017) that show both parameters significantly increased as heating time increased or the food type changed compared to oils before treatment (*p* < .05) (Figures 1, 2).

**TABLE 4:** The effects of independent variables on acid value (variance analysis)

Source	Sum of squares	df	Mean square	*F*	*p*
Corrected model	1741.577	359	4.851	812.765	.000
Intercept	601.104	1	601.104	100708.488	.000
Oil	524.728	3	174.909	29304.164	.000
Time	29.701	3	9.900	1658.699	.000
Temperature	12.791	3	4.264	714.336	.000
Open/close	6.815	4	1.704	285.431	.000
Additive	64.101	2	32.051	5369.723	.000
Error	3.820	640	.006		
Total	2773.390	1000			
Corrected total	1745.397	999			

**TABLE 5:** The effects of independent variables on total polar compounds (variance analysis)

Source	Sum of squares	df	Mean square	*F*	*p*
Corrected model	108,121.948	320	337.881	97.929	.000
Intercept	123,139.701	1	123139.701	35689.912	.000
Additive	10,909.802	1	10909.802	3162.018	.000
Oil	84,886.304	3	28295.435	8200.942	.000
Time	1599.056	3	533.019	154.486	.000
Temperature	1430.278	3	476.759	138.180	.000
Open/close	938.122	4	234.530	67.975	.000
Error	2342.731	679	3.450		
Total	257,141.000	1000			
Corrected total	110,464.679	999			

According to the obtained quantities in terms of resistance to increasing heat, semisolid vegetable oil showed the highest resistance to heat increase compared to frying oil, with a slight difference, followed by cooking liquid oil. The above results are consistent with the study of Sisakhtnejad et al. (2009), which showed the stability of the frying oils available in the Iranian market during frying is low and they cannot be used for frying several times, but in ghee without the presence of food, the AV at the lowest and highest temperatures and times (110°C and 200°C, 5 and 20 min) show at least 1.2% and 4.3%, respectively, which exceeds the allowable limit. Ghee (derived from cow butter) traditionally undergoes a lot of heat during production, in the case of reheating, in most cases higher AV and TPC contents were seen than the standard limit. It indicates that this type of oil should not be used in cooking and even in consumption without heating unless producing under controlled and low temperature condition from the beginning of the production process.

The results showed that the best desirable conditions for the storage of oils were storing in closed containers at refrigerator temperature (5°C). Also, in the case of oils used for frying two types of food, chicken and potato, could be used only for the first time, and in reusing and refrying each one of the foods, chicken and potato, the percentage of TPC and AV go too high and passed off allowed limit, in the other word, they had reached to the disposal point. Yu et al. (2018) reported that TPC content not exceeding rejection limit after 80 times repeated frying that is inconsistent with our results. In similar conditions, oils without the presence of food at a temperature of 110°C and heating time of 20 min, except for ghee samples, were in a long distance to the disposal point, indicating that the role of food in increasing the percentage of TPC and AV is very important. Comparing the TPC and AV contents showed that the oils used for frying of the chicken meat had a higher TPC and AV than the oils used for frying potatoes in all conditions, which may be due to the content of nutrition compounds in chicken meat, such as protein, fat, and their effects on fried oils. Based on statistical analysis of variance (Tables 3, 4), a statistically significant difference was observed in all the oils in terms of oil type, temperature, heating time, storage conditions of the oils (*p* < .05). In other words, the type of oils, temperature, heating time of oils, and their storage conditions for 6 h at room and refrigerator temperatures in open and closed containers were effective in reaching the disposal point.

In order to prevent and reduce chronic diseases and improve general health, targeted lifestyle change programs based on scientific findings should be considered. One of the best ways is to modify the diet including modifying the pattern of oils consumption and continuous education. In addition, it is necessary to monitor the quality of oils consumed in the preparation and distribution centers of ready meals, especially fast foods. To carry out educational, research, and interventional projects with the cooperation of authorities and the staff of preparation and distribution centers, following up and continuous monitoring by environmental health experts of the Health Ministry should be on the agenda.

## 4 CONCLUSION

According to the information obtained from this study, increasing the heating time and temperatures given to the oils lead to the higher AV and TPC contents in the used oils. The increase in the rate of AV and TPC contents of oils was: ghee > cooking liquid oil > frying oil ≥ solid vegetable oil.The results showed that the AV and TPC content of the three desired vegetable oils without the presence of food for frying were within the permissible range at specified temperatures and times in all storage and reheating conditions. But in the case of ghee, in most cases contrary to popular belief, they were above national and international standards. Also, in the process of frying chicken and potato, the reusability of all the studied oils was not possible for the second time and reached the disposal point. The best and most desirable storage conditions were at refrigerator temperature (5°C) and in closed containers. The results show that the oil type and how to use the consumed oils is effective in the AV and TPC rates, and stability and reaching the point of disposal of oils depend on the type of oil, temperature rate, heating time, storage conditions, and heating frequency of the oils.

## CONFLICT OF INTEREST

The authors declare there is no conflict of interest.

## ETHICAL STATEMENT

This study does not involve any human or animal testing.
